# Research ethics in a pandemic: considerations for the use of research infrastructure and resources for public health activities

**DOI:** 10.1093/jlb/lsaa028

**Published:** 2020-05-18

**Authors:** Megan Doerr, Jennifer K Wagner

**Affiliations:** 1 Sage Bionetworks, Seattle, WA 98121, USA; 2 Center for Translational Bioethics & Health Care Policy at Geisinger, Danville, PA 17822, USA

**Keywords:** public health, informed consent, research ethics

## Abstract

The number and size of existing research studies with massive databases and biosample repositories that could be leveraged for public health response against SARS-CoV-2 (or other infectious disease pathogens) are unparalleled in history. What risks are posed by coopting research infrastructure—not just data and samples but also participant recruitment and contact networks, communications, and coordination functions—for public health activities? The case of the Seattle Flu Study highlights the general challenges associated with utilizing research infrastructure for public health response, including the legal and ethical considerations for research data use, the return of the results of public health activities relying upon research resources to unwitting research participants, and the possible impacts of public health reporting mandates on future research participation. While research, including public health research, is essential during a pandemic, careful consideration should be given to distinguishing and balancing the ethical mandates of public health activities against the existing ethical responsibilities of biomedical researchers.

## I. Introduction

Although public health research is undoubtedly essential during a pandemic,^1^ the line between research and public health activities is tricky in the best of times and can blur quickly in a public health emergency.^2^ Elements common to both endeavors range from study design, to the collection and use of personally identifiable and protected health information, and to analysis techniques. Many point to the a priori purpose of a given initiative as a way to distinguish between research and public health activities.^3^ Yet, even while public health practice focuses on assurance, assessment, and policy development, these activities might contribute to generalizable knowledge—the hallmark of research. For example, in 2010 following the Deepwater Horizon oil spill in the Gulf of Mexico, the U.S. Centers for Disease Control and Prevention (CDC) tapped into the National Poison Data System (NPDS) for the purpose of monitoring health impacts of people in the region (ie, surveillance as a public health activity). Nevertheless, the CDC’s utilization of the NPDS post-environmental disaster also demonstrated the database’s utility for advancing scientific understanding of how oil spill exposures affect human health (ie a resources for potential public health research with the primary purpose of contributing generalizable knowledge).^4^ Additionally complicating the divide between research and public health activities^5^ is the now widespread practice of banking of data and samples for secondary research use. During a public health emergency, research repositories are attractive, ready-made data resources and communication channels with large, and, ideally, diverse cohorts through which public health activities could be pursued expeditiously. Given that emergency responses ‘tend to be nonresearch,’^6^ what risks are posed by repurposing *research* infrastructure for public health *activities*?

The COVID-19 pandemic has already provided case examples highlighting key questions about the public health activities that seek to leverage existing research infrastructure. For example, since 2018 the Seattle Flu Study^7^ (SFS) has enrolled research participants and collected nasal swabs with the goal of improving detection, monitoring, and control of influenza outbreaks in greater Seattle, Washington. On March 10, 2020, the *New York Times*^8^ reported on SFS’s ongoing efforts to assess retrospectively the prevalence of the 2019 novel coronavirus, SARS-CoV-2,^9^ using nasal swab samples collected for research purposes during the 2019–20 influenza season. In early February 2020, SFS began petitioning the state, CDC, and U.S. Food and Drug Administration (FDA) officials for permission to use the SFS’s existing sample bank to track COVID-19 spread. SFS participants had consented to the testing of their swabs for influenza and ‘other respiratory pathogens (germs)’ and to receiving these research results back from the study team, as well as for the secondary use of their data for research purposes. Through the consent process, SFS had alerted participants that Washington state law requires reporting of infectious diseases, including influenza,^10^ but did not discuss the use of SFS’s research infrastructure, including data or samples, for other public health activities.^11^ After about 2 weeks of rebuff, and within the context of undeniable national spread of the virus and inadequate testing for it, the SFS team decided to test the samples without the explicit approval of public health authorities or regulators. The SFS team promptly identified a SARS-CoV-2 positive result and alerted local public health officials. The sample was rerun in the Washington state laboratory, where the positive result was confirmed, and the research participant was subsequently notified by public health officials.^12^

Despite this apparent successful use of existing research infrastructure for public health activities,^13^ CDC and FDA regulators ordered SFS to stop retrospective testing of their existing samples immediately but indicated that, with additional consent language clarifying the use of research study materials for public health activities, SFS could prospectively test participants for SARS-CoV-2. In the first few days of March, the University of Washington’s ethical review board determined that, given the public health emergency, SFS had an ethical obligation to test all samples for SARS-CoV-2, citing that SFS already had consent from participants to test for another communicable diseases and return those results and, therefore, was already engaged in both research and public health activities. On March 9, 2020, state regulators again shut down retrospective testing by SFS.^14^ SFS eventually completed its retrospective testing of samples, identifying 25 positive results across 2353 participants, including the first documented case of community transmission of SARS-CoV-2 in the USA.^15^

The back and forth between federal and state authorities, the research team, and the overseeing ethics board, which eventually culminated in the Seattle Flu Study turning its resources toward a joint public health initiative announced March 23, 2020,^16^ illustrates the complexity of the boundary between federally regulated research and public health activities^17^ and highlights key concerns about the repurposing of research infrastructure and its use for public health activities. Firstly, what are the points researchers must consider as they contemplate either mining already collected research data during a public health emergency, or, as in the case of the SFS, undertaking new analyses on already collected samples in the name of public health response? Secondly, what are the considerations for reporting back to research participants types of information derived from public health activities not explicitly disclosed in the informed consent process? Thirdly, given the uncertainty of risks and benefits posed by public health activities, are there any additional concerns raised by legal mandates to disclose information derived from research sources to public health authorities at different governmental levels? These questions are particularly worthy of contemplation given the number of large research initiatives’ data and sample banks that could potentially be called upon by public health authorities during this pandemic—including, notably, the National Institutes of Health’s *All of Us*^SM^ Research Program.

## II. Use of existing research repositories for public health activities

Most federally sponsored human subject research activities are governed by a set of regulations known as the Common Rule.^18^ However, while public health research is governed by the Common Rule, public health activities^19^ are among those deemed ‘not to be research’ and therefore entirely outside of Common Rule’s reach.^20^ This regulatory exception specifically acknowledges that public health activities may ‘use information and biospecimens from a variety of sources,’ including, presumably, from existing research studies or data repositories. Section 46.104(d)(4)(iii) further clarifies consent is not required for secondary use of research data or biospecimens for public health activities. So regardless of whether the data used for public health activities are data that have been previously generated for research or novel data generated from research samples, public health activities are legally considered ‘not research.’ Following from this exemption, the use of research data/specimens for public health activities does not require consent from the individuals to whom those data and samples originated. From this perspective, the SFS would not have needed additional consent of participants for SARS-CoV-2 testing had the SFS’s SARS-CoV-2 testing been designated a public health activity.

Arguments in favor of research data use for public health activities highlight the difference between the profound *physical* and *emotional* harms wrought by historical antecedents, such as the notorious U.S. Public Health Service Syphilis Study at Tuskegee, and the potential *dignitary* harms caused by data or samples previously derived from consented research participants being used for public health activities. And if the primary risk posed to research participants by public health activity use of their data is dignitary harm, researchers should correspondingly consider the privacy rights of participants (outside of those mutually agreed upon in informed consent) before proceeding with these activities.

The most influential health data privacy protections in the U.S. are codified by the Health Insurance Portability and Accountability Act (HIPAA) Privacy Rule.^21^ All covered entities and their ‘business associates’ must follow the HIPAA privacy requirements, which generally covers people/entities providing healthcare, health insurance, or related services. Under HIPAA, outside of their use for care delivery, anyone wanting access to a person’s records must obtain their explicit consent with a few very specific exceptions. One of the exceptions that allows for no-signature release of protected personal health information is the request of a ‘public health authority.’^22^ Within the regulations ‘public health authority’ is broadly conceptualized as a federal, state, or other territorial division’s agency or authority (or their designee), whose mandate includes public health matters.

Notably, the U.S. National Institutes of Health (NIH), the largest funder of biomedical research in the world,^23^ is authorized by law to assist as a ‘public health authority’ based on U.S. Department of Health and Human Services (HHS) interpretation dating back to at least December 2002.^24^ As a public health authority, one might argue that the entirety of the NIH’s research resources—whether NIH-funded researchers or participants are aware or not—might be accessible for use in public health emergencies unless other restrictions would preclude such use. Notably, the SFS, funded through the private Brotman Baty Institute for Precision Medicine,^25^ does not fall under NIH’s public health authority designation and was not designated as a public health activity by state authorities as it initially pursued SARS-CoV-2 testing. Of further interest with regard to privacy protections, during a pandemic, Certificates of Confidentiality—which are shields protecting identifiable sensitive research information from disclosure—are potentially penetrable, as disclosures are permitted if required by laws regarding the reporting of communicable diseases, necessary for the individuals’ medical care, or done with the individuals’ consent.^26^

Although the human subjects research regulations are relatively clear-cut with respect to public health activitie*s*, the ethical considerations for the use of existing research infrastructure for public health activities might not be. Past examples of unethical practice of public health research drove the development of the current regulatory structures intended to protect human research subjects.^27^ Almost two decades ago, Dr Nancy Kass set forth an ethical framework for public health practitioners to assess the implications of public health activities, distinguishing biomedical ethics (which relies heavily upon individual autonomy) and public health ethics (which emphasizes justice, among other principles).^28^ Later Lee, Heilig, and White (2012) provided a compelling justification for the conduct of public health surveillance in the absence of explicit consent from individual patient–participants,^29^ recognizing an ethical obligation to put any public health data collected to use and, similarly, the need to justify nonuse of data that has been collected [‘to use the data we collect for public health benefit; not using the data for improving health must be justified’ (at 42)].

As Felice Batlan highlights in her analysis of national security claims from the lens of public health emergency,^30^ the power to define a ‘public health emergency’ and the ethical concerns raised by these powers are far from straightforward. These complexities are only compounded if individual researchers themselves—rather than designated public health authorities (such as the NIH as a whole) who/which are, at least, politically accountable—take it upon themselves to engage in public health activities, as did the researchers of the SFS who quietly defied state and federal guidance to continue their testing program.^31^ When research resources have been funded by public tax dollars (such as NIH grants), even decisions regarding the well-intentioned donation of supplies and equipment (redirecting such items from research labs that were wound down as nonessential during the pandemic to support emergency medical personnel desperate for personal protective equipment and other supplies) are not easy or straightforward—or necessarily the individual researcher’s decision to make.^32^ Without ethical board oversight or other structured consultation, how are researchers to determine if desired public health activities are a justified use of limited research resources? In the case of the SFS, many have argued that monitoring the outbreak of a novel infectious respiratory disease is an extension of the research team’s existing mandate to study infectious respiratory disease. For a research resource such as the *All of Us*^SM^ Research Program, however, shifting focus (and resources) to a single emerging public health threat might be inconsistent with the central mission. The focus of *All of Us*, honed through unprecedented nationwide community consultation, highlights that ‘unlike research studies that focus on one disease or group of people, *All of Us* is building a diverse database that can inform thousands of studies on a variety of health conditions.’^33^ In this case, researchers might be betraying principles of justice by proposing the use of research resources for a singular public health focus.

Compromising individual rights and interests for public benefit has a fraught and contentious history. Yet even the constitutionally protected right to privacy has long been recognized as not absolute but one that is (i) conditioned upon exercise of that individual right to privacy not interfering with another’s enjoyment of the same right and (ii) subject to reasonable, proportional, and necessary constraints imposed by state and local authorities fulfilling their roles to ensure public health and safety and by federal authorities supplementing such public health responses when they are inadequate.^34^ Moreover, there is a compelling argument that, although not yet widely recognized, there exists a constitutional right to public health.^35^ This argument builds upon an acknowledgment that health has individual and collective aspects, as individuals alone ‘cannot achieve environmental protection, hygiene and sanitation, clean air and surface water, uncontaminated food and drinking water, safe roads and products, or control infectious disease.’^36^ In any case, considering vertical conflicts between local, state, and federal authorities and issues regarding preemption is essential to reconciling researcher obligations that seem to be inconsistent or in conflict within the specific context of a public health emergency. Research repositories that cross jurisdictional boundaries could be particularly complicated in this regard when trying to ensure a uniform research experience as well as equitable distribution of risks and benefits.^37^

## III. Reporting unexpected results

Another dilemma highlighted by the SFS case is the considerations of reporting back to research participants’ information that is not explicitly described in the informed consent process. Further complicating matters in the SFS’s case was the fact that their SARS-CoV-2 test had not, at the time of their original proposal, undergone traditional regulatory review and approval. The majority of the SFS’s laboratories, like many research laboratories, are exempt from the Clinical Laboratory Improvement Amendments of 1988 (CLIA)^38^ and therefore generally are not authorized to return individual research results by the FDA.

The FDA is the oldest consumer protection arm of the federal government and works to ensure that food, drugs, devices, biologics, and others are trustworthy. Nevertheless, since its inception, the FDA has been criticized for ‘slowing the progress’ of medical innovation^39^ and for its perceived political bent.^40^ An Emergency Use Authorization (EUA) under Section 564 of the Federal Food, Drug, and Cosmetic Act (FD&C Act) allows for the special use of unapproved medical products during some types of emergencies.^41^ These are sometimes called ‘medical countermeasures’ (and include, for example, in vitro diagnostic tests, personal protective equipment, antivirals, vaccines, and biological therapeutics) that can be used ‘to diagnose, treat, or prevent serious or life-threatening diseases or conditions’ when there are ‘no adequate, approved, and available alternatives.’^42^ For example, in the case of SARS-CoV-2, HHS Secretary Alex Azar issued a determination on February 4, 2020, that COVID-19 ‘is a public health emergency and that circumstances exist justifying the authorization of emergency use of in vitro diagnostics for detection and/or diagnosis of the novel coronavirus.’^43^ On February 29, 2020, the FDA issued guidance to ‘accelerate the availability novel coronavirus (COVID-19) diagnostic tests developed by laboratories and commercial manufacturers during the public health emergency.’^44^ This guidance stressed the importance of test validation, limits of detection, accuracy, and inclusivity; recommended the inclusion of a transparency statement that the test has been validated but FDA’s independent review of this validation is pending on all results; and required laboratories to report positive results immediately to federal, state, and local public health authorities.^45^

The return of research results has been a Catch-22 for this reason. If researchers share information that turns out to be inaccurate or misleading, they might be held liable for the erroneous disclosure. Alternatively, if researchers withhold information that could be considered clinically relevant, they might be liable for failing to disclose this information. Expert panels^46^ have recommended that research results be returned with clear disclaimers regarding their potential limited reliability and validity, but participants might not fully appreciate these limitations. Liability concerns (at least those related to disclosing the information), however, seem reduced in the context of actions taken in immediate response to COVID-19, given the liability immunity declaration issued by the HHS.^47^ While this immunity declaration unequivocally includes testing for SARS-CoV-2 within its scope of covered countermeasures, researchers do not categorically fall within the scope of covered persons. For immunity protection to be applicable, researchers would need to be recognized as ‘qualified persons.’^48^ It is possible, but not a given, that NIH-funded researchers could be within this category.

Additionally, when considering the return of unexpected research results derived from public health activities, what, if any, considerations should be given to participants right not to know, for example, in the case of SARS-CoV-2 antibody testing? While ‘right not to know’ considerations within the specific context of an oft-fatal infectious disease might seem a stretch, reporting such results might seem contrary to the ‘no surprises’ principle in biomedical research, (which essentially means that researchers should avoid data practices that fail to align with participants’ understanding and expectations).^49^

When asked, the many of participants from a variety of different types of research want and expect to receive results back from their research participation.^50^ Given these expectations, is it necessary to obtain consent to return research results? In the past decade, ‘right not to know’ has been supported primarily in terms of incidental findings on genetic assay.^51^ For many genetic conditions, there are no treatments. However, in the case of results generated as the result of a public health emergency, an individual’s right not to know might be supplanted by the public good of informing them. If research resources are later used for public health activities, a question not definitively answered and likely requiring a case-by-case determination is whether reporting of those results should be treated pursuant to research norms (which historically have required consent) or public health norms (which prioritize information access to control the spread of disease over individual preferences).

Although the return of results might seem like a minor consideration, as ‘back to work’ certificates are being contemplated by many governments, the implications of whether and which SARS-CoV-2 results are to be returned should not be summarily dismissed by researchers or policy makers.^52^ Such concerns underscore the need for a system of ethical board oversight or other structured consultation, to aid researchers in assessing the risks and benefits of using research resources for public health activities.

## IV. Public health reporting

Finally, are there any additional reporting concerns raised by legal mandates to disclose to public health authorities at different governmental levels if consent has not been obtained specifically? Public health reporting varies from aggregate, potentially anonymous data (eg, disease prevalence) to fully identifiable data (eg, contact tracing). Because public health response toolkits include police powers and the ability to infringe upon individual civil liberties, there are understandable concerns regarding the numerous potential uses for research data that might be generated or seized during a public health emergency. For example, because of the immigration law implications (such as the Inadmissibility on Public Charge Grounds final rule), undocumented immigrants might be unwilling to risk seeking health care during the COVID-19 pandemic regardless of public statements from U.S. Citizenship and Immigration Services (within the Department of Homeland Security) that seeking services to test, treat, or prevent COVID-19 would ‘not negatively affect’ any individual in the Public Charge analysis.^53^ The inclusivity of a research data set being contemplated for use as part of a response during a public health emergency might require careful consideration regarding whether doing so advances or impedes an equitable distribution of the benefits and risks not only of the public health surveillance itself but also (i) the actions taken and policies developed and implemented based on those results made possible with that research resource and (ii) the subsequent willingness to participate in research.^54^

One example to highlight this dilemma is contact tracing. Public health authorities in other nations have adopted contact tracing to identify networks of exposed people.^55^ Given that human subject research studies now commonly include connected devices which collect data that could be valuable in contact tracing, this is of particular concern. Researchers themselves struggle with appreciating the scope and implications of privacy concerns raised by the scope of big data research, ^56^ leaving ethics review boards and the participants they serve at a loss.^57^ In the public health emergency context, these powerful data might only further obscure variables in the delicate calculus of individual risk and public benefit, underscoring the benefit of establishing formal consultation and review processes for public health activities that would use research data.

## V. Conclusion

Both the volume and granularity of data collected in research repositories are orders of magnitude greater than it has ever been. However, utilizing these data—as well as the research infrastructure that supports them—in the name of public health response is not without risk. The differing legal and ethical obligations for research and public health activities are worthy of researchers’ careful consideration even in the face of a public health emergency imposing powerful urgency constraints on decision-making. To be clear, these tensions should not inhibit research from proceeding during a pandemic nor the transfer of research resources to public health activities per se. Rather, it is incumbent upon the research community, including biomedical legal and ethical scholars and practitioners, to reflect upon the many tensions experienced during the COVID-19 pandemic between public health initiatives (the infrastructure and support for which has been proven woefully inadequate in the U.S.) and biomedical research (the leveraging of which might be particularly useful in times of public health emergencies, regardless of the state of public health infrastructure) and consider the creation of a formal consultative process, so that, in the future, research infrastructure might be called upon both responsibly and swiftly to augment public health initiatives. Further, as ever larger and more diverse datasets are amassed, the lines between research and public health activities—not to mention clinical care—will continue to blur. The current pandemic highlights the need for each of these communities—researchers, public health authorities, and clinicians—to reconsider the legal and ethical bounds of their mandates and critically examine areas of overlap. Active engagement with policy makers is needed. Finally, it would be particularly prudent for the research community, equipped with its robust resources and good intentions, to think critically about how to avoid the research enterprise being simply an enabler for the continued neglect of public health in the U.S.

## Footnotes

1 Kayte Spector-Bagdady, *The Importance of Doing Research during a Pandemic*, USA Today, Mar. 19, 2020, https://www.usatoday.com/story/opinion/2020/03/19/coronoavirus-importance-doing-research-during-pandemic-column/5043852002/ (accessed Mar. 28, 2020).

2 Eg World Health Organization, *Ethical Standards for Research during Public Health Emergencies: Distilling Existing Guidance to Support COVID-19 R&D*, 2020, https://www.who.int/blueprint/priority-diseases/key-action/liverecovery-save-of-ethical-standards-for-research-during-public-health-emergencies.pdf?ua=1 (accessed Apr. 8, 2020); Biobot Analytics, *How Many People are Infected with Covid-19? Sewage Suggests that Number is much Higher than Officially Confirmed,* Medium, Apr. 8, 2020, https://medium.com/@biobotanalytics/how-many-people-are-infected-with-covid-19-e7e8c6195d6b (accessed Apr. 9, 2020).

3 Jean Lin Otto, Mark Holodniy & Robert F. DeFraites, *Public Health Practice is Not Research*, 104 Am. J. Public Health 596 (2014); James G. Hodge, *Distinguishing Public Health Practice and Human Subjects Research: A White Paper for the University of Virginia Institute for Practical Ethics and Public Life*, http://dph.illinois.gov/sites/default/files/resources/resources-irb/whitepaper-practice-and-research-041516.pdf (accessed Apr. 9, 2020).

4 Law R, Martin C, Wolkin A, et al., *Use of the National Poison Data System for Surveillance of Human Health Effects from the Deepwater Horizon Oil Spill*. 4 Emerg. Health Threats J. 27 (2011).

5 For general discussion, see “Research and Public Health Emergencies,” *Sage Assembly Webinar Series*, April 9, 2020, https://youtu.be/DTIsVuOEo-g (accessed Apr. 11, 2020) (wherein panelists discussed, among other topics, the similarities of the public health activity/research dichotomy and the intentionally blurred dichotomy of research and care within a learning healthcare system).

6 Eg C.D.C., *“Distinguishing Public Health Research and Public Health Nonresearch” Policy*, CDC-SA-2010-02, (2010) at 5, https://www.cdc.gov/od/science/integrity/docs/cdc-policy-distinguishing-public-health-research-nonresearch.pdf (accessed May 6, 2020).

7 Seattle Flu Study, https://seattleflu.org/ (accessed Mar. 28, 2020).

8 Sheri Fink & Mike Baker, *‘It’s Just Everywhere Already’: How Delays in Testing Set Back the U.S. Coronavirus Response*, N.Y. Times, Mar. 10, 2020, https://www.nytimes.com/2020/03/10/us/coronavirus-testing-delays.html (accessed Mar. 28, 2020); also Sheri Fink & Mike Baker, *A Lab Pushed for Early Tests, But Federal Officials Said No,* N.Y. Times, Mar. 11, 2020, § A, at 1.

9 Martin Enserink, *Update: ‘A bit chaotic.’ Christening of New Coronavirus and its Disease Name Create Confusion.*Science, Feb. 12, 2020, https://www.sciencemag.org/news/2020/02/bit-chaotic-christening-new-coronavirus-and-its-disease-name-create-confusion (accessed Mar. 24, 2020).

10 Wash. Admin. Code § 246-101-525 (2019), https://app.leg.wa.gov/wac/default.aspx?cite=246-101-525 (accessed Mar. 28, 2020).

11 Seattle Flu Study, *Privacy Highlights*, Nov. 1, 2019, https://seattleflu.org/privacy (accessed Apr. 9, 2020).

12 Allison Sundell, *New Coronavirus Cases Confirmed in Snohomish, King Counties*, Feb. 28, 2020, https://www.king5.com/article/news/health/coronavirus/washington-coronavirus-update/281-e73682dc-dad7-4b6e-b0ec-d2234ff9e2e0 (accessed May 7, 2020).

13
https://twitter.com/trvrb/status/1233970271318503426 (accessed May 8, 2020).

14
https://twitter.com/trvrb/status/1237612761263140864 (accessed May 8, 2020) and https://twitter.com/seattleflustudy/status/1237601606037721088 (accessed May 8, 2020).

15 Helen Y. Chu et al., *To the Editor: Early Detection of Covid-19 Through a Citywide Pandemic Surveillance Platform*, N. Eng. J. Med., May 1, 2020, https://www.nejm.org/doi/full/10.1056/NEJMc2008646.

16 Seattle Flu Study, *Leadership Update from the Seattle Flu Study*, https://seattleflu.org/scan (accessed Apr. 11, 2020); SCAN: Greater Seattle Coronavirus Assessment Network, https://scanpublichealth.org/ (accessed Apr. 11, 2020); https://publichealthinsider.com/2020/03/23/introducing-scan-the-greater-seattle-coronavirus-assessment-network/ and https://twitter.com/trvrb/status/1242136969922015235.

17 See, eg Jean Lin Otto, Mark Holodniy & Robert F. DeFraites, *Public Health Practice is Not Research*, 104 Am. J. Public Health 596 (2014); Lester Darryl Geneviève et al., *Participatory Disease Surveillance Systems: Ethical Framework*, 21 J. Med. Internet Res. e12273 (2019).

18 Final Rule: Federal Policy for the Protection of Human Subjects. 82 Fed. Reg. 7149 (Jan. 19, 2017), codified at 45 C.F.R. § 46.

19 45 C.F.R. § 46.102(l)(2).

20 U.S. Dept. of Health and Human Services, Office for Human Research Protections, *Activities Deemed Not to Be Research: Public Health Surveillance 2018 Requirements* (reviewed Nov. 12, 2018), https://www.hhs.gov/ohrp/regulations-and-policy/requests-for-comments/draft-guidance-activities-deemed-not-be-research-public-health-surveillance/index.html (accessed Mar. 28, 2020).

21 Health Information Portability and Accountability Act of 1996, Pub. L. 104-191, 110 Stat. 1936, as codified at 45 C.F.R. §§ 160, 162, and 164.

22 45 C.F.R. § 164.512(b).

23 Robin Seaton Jefferson, *How the Largest Public Funder of Biomedical Research in the World Spends Your Money*, Forbes, Dec. 21, 2018, https://www.forbes.com/sites/robinseatonjefferson/2018/12/21/how-the-largest-public-funder-of-biomedical-research-in-the-world-spends-your-money/#430487f227b9 (accessed Mar. 28, 2020).

24 U.S. Dept. of Health and Human Services, *Does the HIPAA Privacy Rule’s Public Health Provision Permit Covered Entities to Disclose Protected Health Information to Authorities such as the National Institute of Health (NIH)?*, Dec. 12, 2002, https://www.hhs.gov/hipaa/for-professionals/faq/297/does-the-hipaa-public-health-provision-permit-covered-entities-to-disclose-information-to-authorities/index.html (accessed Mar. 28, 2020).

25
https://www.medrxiv.org/content/10.1101/2020.03.02.20029595v1.full.pdf.

26 Eg National Institutes of Health, *What is a Certificate of Confidentiality?*, Jan. 14, 2019, https://grants.nih.gov/policy/humansubjects/coc/what-is.htm (accessed Apr. 8, 2020).

27 Centers for Disease Control and Prevention, *U.S. Public Health Service Syphilis Study at Tuskegee* (reviewed Mar. 2, 2020), https://www.cdc.gov/tuskegee/index.html (accessed Mar. 28, 2020); Michael A. Rodriguez & Robert García, *First Do No Harm: The US Sexually Transmitted Disease Experiments in Guatemala*, 103 Am. J. Public Health 2122 (2013), https://www.ncbi.nlm.nih.gov/pmc/articles/PMC3828982/.

28 Nancy E. Kass, *An Ethics Framework for Public Health*, 91 Am. J. Public Health 1776 (2001).

29 Lisa M. Lee, Charles M. Heilig & Angela White, *Ethical Justification for Conducting Public Health Surveillance Without Patient Consent*, 102 Am. J. Public Health 38 (2012).

30 Felice Batlan, *Law in the Time of Cholera: Disease, State Power, and Quarantines Past and Future*, 80 Temple L. Rev. 53 (2007), https://ssrn.com/abstract=1719144, https://papers.ssrn.com/sol3/papers.cfm?abstract_id=1719144.

31 Charles Duhigg, *Seattle’s Leaders Let Scientists Take the Lead. New York’s Did Not*, The New Yorker, Apr. 26, 2020, https://www.newyorker.com/magazine/2020/05/04/seattles-leaders-let-scientists-take-the-lead-new-yorks-did-not (accessed May 8, 2020).

32 Countless examples of individual researcher efforts and coordinated efforts at various academic institutions to donate research supplies are available. Eventually, once the dust settles on the COVID-19 pandemic, a reckoning as to whether, under what circumstances, and to what extent the leveraging of research resources for non-research purposes in response to COVID-19 constitutes fraud, abuse, or waste. Researchers might find themselves ill-equipped to answer these questions alone, and it is unclear whether institutions have had adequate oversight over redistributions of supplies and equipment.

33 NIH, *All of *Us* Research Program Overview*, https://allofus.nih.gov/about/all-us-research-program-overview (accessed May 8, 2020).

34 See, eg Lawrence O. Gostin & James G. Hodge, Jr., *The Public Health Improvement Process in Alaska: Toward A Model Public Health Law*, 17 Alaska L. Rev. 77 (2000); Pekham Pal, *History, Governmental Structure, and Politics: Defining the Scope of Local Board of Health Power*, 84 Fordham L. Rev. 769 (2015); Lawrence O. Gostin, James G. Hodge, Jr. & Lindsay F. Wiley, *Presidential Powers and Response to COVID-19*, JAMA, Mar. 18, 2020, https://jamanetwork.com/journals/jama/fullarticle/2763423 (accessed Apr. 8, 2020).

35 James G. Hodge, Jr. et al., *Constitutional Cohesion and the Right to Public Health*, 53 U. Mich. J. L. Reform 173 (2019).

36
* Id.* at 222 (quoting in FN315 Lawrence O. Gostin, *Mapping the Issues: Public Health, Law and Ethics*, in Public Health Law and Ethics: A Reader 2 (2nd ed. 2010)) (emphasis added). The interaction of and relationship between the right of privacy and right of public health are both interesting and important considerations; however, given such a discussion requires advanced legal analysis and involves complex legal theory, it has been left for discussion elsewhere.

37 See also Megan Doerr et al., *Implementing a Universal Informed Consent Process for the All of Us Research Program*, 24 Pac. Symp. Biocomput. 427 (2019).

38 42 C.F.R. § 493.

39 Eg Nurith Aizenman, *How to Demand a Medical Breakthrough: Lessons From the AIDS Fight*, NPR, Feb. 9, 2019, https://www.npr.org/sections/health-shots/2019/02/09/689924838/how-to-demand-a-medical-breakthrough-lessons-from-the-aids-fight (accessed Mar. 28, 2020).

40 Eg Sarah Jane Tribble, *FDA Moves to Rein in Drugmakers’ Abuse of Orphan Drug Law*, NPR, Sept. 13, 2017, https://www.npr.org/sections/health-shots/2017/09/13/550700062/fda-moves-to-rein-in-drugmakers-abuse-of-orphan-drug-law (accessed Mar. 28, 2020).

41 Federal Food, Drug, and Cosmetic Act, Pub. L. 75-717, 52 Stat. 1040, 21 U.S.C. § 301–392, as amended to add Section 564 by the Project BioShield Act of 2004, Pub. L. 108-276, and as amended by 21^st^ Century Cures Act, Pub. L. 113-5, 21 U.S.C. 360bbb-3, 360bbb-3a, and 360bbb-3b. For example, during the 2009 H1N1 pandemic, the FDA issued an EUA so an unapproved antiviral drug could be used as a treatment, but the drug was not ultimately approved until 2014. See Authorization of Emergency Use of the Antiviral Product Peramivir Accompanied by Emergency Use Information; Availability, 74 Fed. Reg. 56644 (Nov. 2, 2009) and James G. Hodge, Jr., Lexi White & Sarah Wetter, *From (a)nthrax to (z)ika: Key Lessons in Public Health Legal Preparedness*, 15 Ind. Health L. Rev. 23, 40 (2018).

42 Eg DHHS, FDA, Office of the Commissioner, Office of the Chief Scientist, Office of Counterterrorism and Emerging Threats, *Emergency Use Authorization of Medical Products and Related Authorities: Guidance for Industry and Other Stakeholders*, FDA-2016-D-1025, Jan. 2017, at 2–3, https://www.fda.gov/regulatory-information/search-fda-guidance-documents/emergency-use-authorization-medical-products-and-related-authorities#euas (accessed Mar. 28, 2020).

43 HHS Secretary Alex M. Azar, *Determination of a Public Health Emergency and Declaration that Circumstances Exist Justifying Authorizations Pursuant to Section 564 (b) of the Federal Food, Drug, and Cosmetic Act, 21 U.S. C. § 360bbb-3*, Feb. 4, 2020, https://www.fda.gov/media/135010/download (accessed Mar. 28, 2020).

44 FDA, Center for Devices and Radiological Health, *Policy for Diagnostic Tests for Coronavirus Disease-2019 during the Public Health Emergency: Immediately in Effect Guidance for Clinical Laboratories, Commercial Manufacturers, and Food and Drug Administration Staff*, Mar. 16, 2020, https://www.fda.gov/media/135659/download, at 1 (accessed Mar. 28, 2020).

45
*Id.* at 4.

46 National Academies of Sciences, Engineering, and Medicine, Returning Individual Research Results to Participants: Guidance for a New Research Paradigm, (Autumn S. Downey, Emily R. Busta, Michelle Mancher and Jeffrey R. Botkin eds, National Academies Press, 2018).

47 Declaration Under the Public Readiness and Emergency Preparedness Act for Medical Countermeasures Against COVID-19, 85 Fed. Reg. 15198, (Mar. 17, 2020), https://www.federalregister.gov/documents/2020/03/17/2020-05484/declaration-under-the-public-readiness-and-emergency-preparedness-act-for-medical-countermeasures (accessed May 7, 2020).

48 Defined by the Declaration, *Id.* at 15201-2, as ‘(a) Any person authorized in accordance with the public health and medical emergency response of the Authority Having Jurisdiction...; (b) any person authorized...to perform an activity under an Emergency Use Authorization...; and (c) any person authorized to prescribe, administer, or dispense Covered Countermeasures in accordance with Section 564A of the FD&C Act.’

49 See, eg Dixie B. Baker, Jane Kaye & Sharon F. Terry, *Governance through Privacy, Fairness, and Respect for Individuals,* 4 eGEMs 1207 (2016) (explaining the ‘no surprises principle’ as ‘assert[ing] that an individual’s personal information should never be collected, used, transmitted, or disclosed in a way that would surprise the individual were she to learn about it.’).

50 Eg Christopher R. Long, M. Kathryn Stewart & Pearl A. McElfish, *Health Research Participants are not Receiving Research Results: A Collaborative Solution is Needed*, 18 Trials 449 (2017); Christopher R. Long et al., *Health Research Participants’ Preferences for Receiving Research Results*, 13 Clin. Trials 582 (2016).

51 Roger Brownsword & Jeff Wale, *The Right to Know and the Right Not to Know Revisited: Part One*, 9 Asian Bioethics Rev. 3 (2017).

52 Henry T. Greely, *Covid-19 ‘Immunity Certificates’: Practical and Ethical Conundrums*, STAT News, Apr. 10, 2020, https://www.statnews.com/2020/04/10/immunity-certificates-covid-19-practical-ethical-conundrums/ (accessed May 7, 2020); Mark A. Hall & David M. Studdert, *Privileges and Immunity Certification During the COVID-19 Pandemic*, JAMA (2002), https://jamanetwork.com/journals/jama/fullarticle/2765835 (accessed May 7, 2020); Kate Proctor, Ian Sample & Philip Oltermann, *‘Immunity Passports’ could Speed up Return to Work after Covid-19*, The Gaurdian, Mar. 30, 2020, https://www.theguardian.com/world/2020/mar/30/immunity-passports-could-speed-up-return-to-work-after-covid-19 (accessed May 7, 2020); Michelle Roberts, *Coronavirus: Scientists Publish Advice to Government,* BBC News, May 5, 2020, https://www.bbc.com/news/health-52542109 (accessed May 7, 2020).

53 Kathleen R. Page et al., *Undocumented U.S. Immigrants and Covid-19*, New Eng. J. Med. (2020), https://www.nejm.org/doi/full/10.1056/NEJMp2005953 (accessed Apr. 9, 2020).

54 See, eg Jennifer K. Wagner, *Ethical and Legal Considerations for the Inclusion of Underserved and Underrepresented Immigrant Populations in Precision Health and Genomic Research in the US,* 29 Ethnic. Dis. 641 (2019).

55 Allison Kaplan Sommer, *Israel Unveils Open Source App to Warn Users of Coronavirus Cases,* Harretz, Mar. 23, 2020, https://www.haaretz.com/israel-news/israel-unveils-app-that-uses-tracking-to-tell-users-if-they-were-near-virus-cases-1.8702055 (accessed Apr. 8, 2020); Min Joo Kim & Simon Denyer, *A ‘Travel Log’ of the Times in South Korea: Mapping the Movements of Coronavirus Carriers*, The Washington Post, Mar. 13, 2020, https://www.washingtonpost.com/world/asia_pacific/coronavirus-south-korea-tracking-apps/2020/03/13/2bed568e-5fac-11ea-ac50-18701e14e06d_story.html (accessed Apr. 8, 2020); Saheli Roy Choudhury, *Singapore Says It Will Make Its Contact Tracing Tech Freely Available to Developers,* CNBC, Mar. 25, 2020, https://www.cnbc.com/2020/03/25/coronavirus-singapore-to-make-contact-tracing-tech-open-source.html (accessed Apr. 8, 2020).

56 Barbara L. Filkins et al., *Privacy and Security in the Era of Digital Health: What should Translational Researchers Know and Do About It?,* 8 Am J Transl Res. 1560 (2016); Elias Chaibub Neto et al., *Detecting the Impact of Subject Characteristics on Machine Learning-based Diagnostic Applications.* 2 NPJ Digit Med. 99 (2019).

57 Cinnnamon Bloss et al., *Reimagining Human Research Protections for 21st Century Science*, 18 J. Med. Internet Res. e329 (2016).

